# Bereaved parents’ perceptions of their cancer-ill child’s last month with or without palliative care - a nationwide study

**DOI:** 10.3389/fonc.2024.1387905

**Published:** 2024-10-22

**Authors:** Margaretha Stenmarker, Lilian Pohlkamp, Josefin Sveen, Ulrika Kreicbergs

**Affiliations:** ^1^ Department of Pediatrics, Institute of Clinical Sciences, Sahlgrenska Academy, University of Gothenburg, Gothenburg, Sweden; ^2^ Department of Pediatrics, Region Jönköping County, Linköping, Sweden; ^3^ Department of Biomedical and Clinical Sciences, Linköping University, Linköping, Sweden; ^4^ Department of Caring Sciences, Marie Cederschiöld University, Stockholm, Stockholm, Sweden; ^5^ Centre for Crisis Psychology, Faculty of Psychology, University of Bergen, Bergen, Norway; ^6^ University College London, London, United Kingdom

**Keywords:** pediatric oncology, palliative care, children, adolescent, cancer, palliative cancer care, parents, bereaved parents

## Abstract

**Background:**

Cancer is still the leading cause of non-accidental death in childhood, although the majority of children diagnosed in high-income countries survive their illness. In accordance with international standards, equal and early access to palliative care should be available to children and adults. Yet communication and prognostic disclosure may influence the timing of involvement in palliative care. Purpose: To investigate whether parents perceived that their child received palliative care and to what extent that contrasted parents’ perceptions of their child’s care and symptoms in the last month of life.

**Methods:**

A nationwide population-based parental questionnaire study in Sweden, one to five years after their child’s death (n=226). Descriptive statistics were used.

**Results:**

A majority of parents (70%) reported that they were aware that their child received palliative care and they were informed about the incurable disease (57%) within 3 months before the child died. The most common diagnosis among children receiving palliative care was a brain tumor (45%) with a disease related death (90%) and the care was often received at home (44%). Based on the reports of parents who felt that their child did not receive palliative care, 45% were informed within days or hours about the child’s incurable disease, 45% of these children were diagnosed with leukemia, 60% died at the intensive care unit, and 49% died of treatment-related complications. It was most common for families who lived in urban areas (28%) to report their child received palliative care, in comparison to families living in sparsely populated areas (11%). A significant proportion of parents whose child received palliative care (96%) stated that the healthcare professionals were competent in caring for their child, for those who reported no palliative care it was slightly lower (74%). In both groups many children were affected by multiple symptoms the last month of life.

**Conclusions:**

The study findings highlight the role of understanding parental perceptions of pediatric palliative oncology care, the role of initiating palliative care early, the need of access to national equitable PC and professional competence across the lifespan, regardless of diagnosis and place of residence.

## Introduction

Worldwide, approximately 400 000 children and adolescents aged 0 to 19 are diagnosed with cancer every year (1). Childhood cancer is an example of the group of diseases where a majority of children diagnosed in high-income countries survive their illness today, although survival varies between different diagnoses (2). The cancer-trajectory can be prolonged, the treatment can be intense, and it is not uncommon for the treatment to pose a threat to the child’s well-being and even their life (2). The definition of pediatric palliative care, according to the World Health Organization (WHO) in 1998, was based on experiences of caring for children with cancer. Today, the definition includes all diagnoses and emphasizes the role of active multidimensional care that provides support to all family members and carried out with interdisciplinary competence (3). The World Health Organization recommends that palliative care begins when a life-threatening disease or a life-limiting condition is diagnosed, and continues regardless of whether the child receives targeted treatment or not (3). The International Association for Hospice and Palliative Care (IAHPC) has further developed the WHO definition to clearly encompass all ages, both children and adults. Most importantly, this proposal focuses on the suffering of a person with a severe illness, rather than the life-threatening illness itself (4). In 2007, standards for pediatric palliative care were presented by an international consortium of experts and these standards have recently been updated (5). The threat of losing a child is a profound trauma for parents (6). Regardless of the diagnosis, life changes for the entire family when a child is diagnosed with a life-threatening illness (7). Parents struggle to grasp the information (8) and handle the situation, irrespective of prognosis (9). Research has shown that parents want information in as much detail as is possible, even when the prognosis is uncertain or the disease is incurable (10–12). The timing of conversations imparting bad news is crucial. Sometimes parents are informed too late, or not informed at all, that their child might not survive (11).

In Sweden, around 350 children and adolescents (under 19 years old) are diagnosed with cancer each year, and approximately 60 die from the disease or treatment-related complications. The Swedish healthcare model has a decentralized structure with 21 county councils, is tax-financed and different healthcare services provide palliative care. The care is based on a regional and local organization, including specialized and primary palliative caregivers (13, 14). The majority of children in need of palliative care have access to specialized services; however, Sweden has only one hospice for children. This means that there is varying access to pediatric palliative care and specialists within the field. To align with international standards for pediatric palliative care, underlining children’s right to equal care, national and regional initiatives are ongoing (15). Yet, access to palliative care is not solely a matter of organization. Individual knowledge and attitudes toward palliative care among healthcare professionals may influence communication regarding palliative care and the time of involvement. Many care providers still see palliative care as the very last option, and then the benefits may not be fully experienced by their patients. Thus, the aim of the current nationwide study was to investigate whether parents perceived that their child received palliative care and to what extent this contrasted parents’ perceptions of their child’s care and symptoms in the last month of life.

## Materials and methods

### Design

This was a nationwide population-based questionnaire study conducted with parents one to five years after their child’s death due to cancer, evaluating the end-of-life care.

### Study population

In 2016, presumptive study participants were identified based on three Swedish national quality registries, which all have a very high coverage rate. Since the early 1980s, children diagnosed with cancer have been registered in the Swedish Childhood Cancer Registry. This national register was used to identify children diagnosed with cancer before the age of 17 and during the years 2010-2015. Register data were linked to the Cause of Death Registry, established in 1961, which reports all children and young adults who had died from childhood cancer. Parents of the deceased children were identified through the Swedish Population Register at the Swedish Tax Agency, established in its current form in 1946. This identification was possible based on the child’s personal identification number.

### Study-specific questionnaire

The questionnaire was developed with items based on previous research (16) and sociodemographic data. A validation process was performed to ensure that the items were interpreted by the participants as intended by the researchers and to guarantee quality and relevance to the targeted population. The validation consisted of face- to-face interviews, conducted by the second author, with parents who had lost a child to cancer. The variables included in this study present data on parents’ awareness of the healthcare their child received, i.e., whether the child received palliative care or not, the parental view of aspects of healthcare, and the child’s symptoms during the last month of life. Previous publications based on the same national survey present the bereaved parents’ perceived social support, levels of prolonged grief, their psychological health, and the role of family communication (10, 17–20).

### Procedure

An introductory letter was sent to each identified registered parent (n=512) of the deceased child. The letter described the study purpose, design, and procedure and provided contact information for the research group. In the questionnaire, the research group added information regarding the definition of palliative care, namely, ‘Palliative care refers to symptom-relieving care provided without the direct intention to cure’. This was done to facilitate the parent’s accurate response to the study’s central question of whether their child had received palliative care. Approximately ten days later, all parents were contacted by telephone and asked if they would consent to participate. If there was a lack of information concerning the parents’ telephone numbers, letters were sent to their home addresses requesting them to contact the research group. Upon receiving positive consent, a questionnaire was sent by mail with a prepaid return envelope. No questionnaires were sent without consent. Around two weeks later, a reminder call was made to those who did not return the questionnaire.

The Regional Ethical Review Board in Stockholm, Sweden, approved the study (No. 2015/2183-31/5)

### Statistics

Descriptive statistics are presented as frequencies, percentages, mean values, and standard deviations. Comparisons between responders and non-responders regarding sociodemographic data were performed using the Wilcoxon rank-sum test and chi-square analyses. The significance level was set to P < 0.05. All data were processed confidentially with code numbers. If a questionnaire contained more than 10% missing data, it was excluded from further analysis. All statistical analyses were performed using the Statistical Package for the Social Sciences for Windows, version 22 (SPSS, Chicago, IL).

## Results

In total, 530 bereaved parents were eligible in the registries, of which 157 were excluded. The reasons for exclusion were as follows: they could not be reached by e-mail or by telephone (n=76), they declined participation (n=63) or they did not have sufficient knowledge of Swedish to answer the questionnaire (n=18). Questionnaires were sent to all bereaved parents (each parent separately in case of the same child) who consented to participate (n=373) and those who returned the questionnaires were included (n= 232). The study population was divided into three groups based on the following question: did your child receive palliative care? In total the question was answered by 226 bereaved parents and 155 children are represented by the study, i.e., 71 children are reported by two parents. The concordance between parents was high and equal to 93%. The majority of the participating parents were married or lived with a partner and were employed. The total study population of parents is presented in **Table 1**.

**Table 1:** Parental demographic data and factors related to the parent’s perception regarding the provided information and the child’s care.

	Yes, my child received PC, n (%)	No, my child did not receive PC, n (%)	I am uncertain if my child received PC, n (%)
**Did your child receive palliative care (PC)?**	160 (70)	47 (21)	19 (9)
**Parent mean age (SD) at child death**	42.7 (7.86)	40.3 (7.30)	43.0 (10.56)
Parent sex			
Female	93 (58)	27 (57)	13 (68)
Male	67 (42)	20 (43)	6 (32)
Marital status			
Married/living with a partner	146 (92)	41(87)	15 (79)
Living apart (but as a couple)	2 (1)	1 (2)	1 (5)
Single	12 (8)	4 (9)	3 (16)
*Missing*	-	1 (2)	-
Main occupation			
Employed	134(84)	35(74)	15(79)
Studying	2 (1)	2 (4)	-
Parental leave	13 (8)	8 (17)	2 (11)
Unemployed	4 (3)	-	1 (5)
Sick leave	3 (2)	1 (2)	-
House	3 (2)	–	1 (5)
*Missing*	1 (1)	1 (2)	-
Residential region			
Sparsely populated area	17 (11)	8 (17)	4 (21)
Rural area	41 (26)	20 (43)	4 (21)
Town	24 (15)	7 (15)	5 (26)
City	31 (19)	6 (13)	1 (5)
Urban area	45 (28)	5 (11)	4 (21)
*Missing*	2 (1)	1 (2)	1 (5)
How did you get the information that your child’s illness was incurable?			
With my child and the other parent	48 (30)	6 (13)	3 (16)
With the other parent	78 (49)	12 (25)	8 (42)
With my child without the other parent	5 (3)	–	–
I was alone	15 (9)	1 (2)	5 (26)
Other ways	7 (4)	3 (6)	1 (5)
I never got such information	6 (4)	25 (53)	2 (10)
*Missing*	1 (1)	–	–
How long before your child died did you get the information that the illness was incurable?			
Within 24 hours	4 (3)	13 (28)	3 (16)
A couple of days before	10 (6)	8 (17)	1 (5)
A week before	8 (5)	3 (6)	1 (5)
2-4 weeks before	31 (19)	–	3 (16)
1-3 months before	38 (24)	2 (4)	3 (16)
4-6 months before	23 (14)	1 (2)	3 (16)
7-11 months before	18 (11)	-	–
One year or longer before	23 (14)	-	1 (5)
I never got such information	5 (3)	19 (40)	2 (11)
*Missing*	-	1 (2)	2 (11)
Could you assimilate the information that your child’s illness was incurable?			
Completely agree	58 (36)	9 (19)	5 (26)
Mostly agree	55 (34)	10 (21)	9 (47)
Slightly agree	35 (22)	9 (19)	0
Completely disagree	10 (6)	6 (13)	5 (26)
*Missing*	2 (1)	13 (28)	-
Where did your child receive PC? *If yes, multiple options are possible*			
Pediatric Oncology Center	55 (34)	–	8 (42)
At a county hospital	109 (68)	1 (2)	12 (63)
At home with home care	68 (42)	1 (2)	10 (53)
At home without home care	138 (86)	–	13 (68)
Other form of care	137 (86)	–	12 (63)
*Missing*	–	–	–
For how long time did your child receive PC?			
Some days	35 (22)		6 (43)
Some weeks	36 (23)		2 (14)
Around one month	41 (26)		4 (29)
Several months up to one year	35 (22)		2 (14)
One year or more	9 (6)		-
*Missing*	4 (3)		5 (27)
Where did your child die?			
At the intensive care unit	14 (9)	28 (60)	4 (21)
At the pediatric oncology center	13 (8)	3 (6)	4 (21)
At a county hospital	28 (18)	7 (15)	2 (11)
At home	70 (44)	4 (9)	3 (16)
In another form of care	16 (10)	3 (6)	1 (5)
*Missing*	19 (12)	2 (4)	5 (26)

The study material was analyzed and is presented in relation to the parental answer, i.e., those who answered ‘yes’, those who answered ‘no’, and those who were uncertain if their child had received palliative care or not. In a dropout analysis, comparing those who responded to the survey and those who did not respond, there were no significant differences regarding the sociodemographic data presented in **Table 1**. However, the percentage of women was significantly higher among responders in comparison with non-responders (x^2^ = 8.83; P <0.05).

### Factors related to the child’s care

A majority of the parents (70%) reported that their child had received palliative care, 21% reported that their child did not receive palliative care and 9% were uncertain whether their child had received palliative care or not. In the group of parents who reported that their child received palliative care, 9% were informed that the disease was incurable within hours to days before the child’s death, while 3% were not informed at all. Corresponding numbers for parents who reported that their child did not receive palliative care were, 45% and 40%, respectively. The majority of parents who reported that their child received palliative care were informed about the child’s incurable disease with the other parent present (49%), and 30% with both the child and the other parent present. The majority (92%) of parents who answered ‘yes’ to the question that palliative care was received stated that they were able to assimilate the message that the disease was incurable, in comparison with 59% among those who answered ‘no’ and 73% of those who were uncertain. If palliative care was received, the most common place of care was at home without home care (86%) and the least common place was a pediatric oncology center (34%). The duration of palliative care varied from a few days, weeks, or several months up to a year, with roughly equal reported percentages for each time interval. A smaller proportion (6%) received palliative care for a year or longer. Among children receiving palliative care, most died at home (44%), in comparison with no reported palliative care where 9% died at home and 60% died at the intensive care unit (**Table 1**).

### Factors related to the parent and the parental perception of the child’s care during the last month of life

Parents of children who received palliative care perceived that they had influence in the child-care-related decisions (93%), received a quick response from healthcare professionals when assistance was needed (96%), and, based on the individual needs of the child, hospital (87%) or home (78%) care was provided. Similar professional support and guidance were experienced by parents in the groups ‘No, my child did not receive palliative care’ and ‘I am uncertain if my child received palliative care’. It was most common for families who lived in urban areas (28%) to report their child received palliative care, followed by rural areas (26%), cities (19%), towns (15%), and sparsely populated areas (11%). In the ‘No, my child did not receive palliative care’ group the majority lived in rural areas (43%). A significant proportion of caregivers in all three groups, ‘Yes, my child received palliative care’ (96%), ‘No, my child did not receive palliative care’ (74%) and those who were uncertain (89%), reported that the healthcare professionals were competent in caring for their child.

Based on the parental report, the parents played a significant role in the healthcare during the final month. The majority with a child receiving palliative care participated in their child’s care throughout the entire illness trajectory (n=134, 84%), and many felt that they were involved to the extent that they desired (n=116, 73%). More than half of the parents (62%) whose child received palliative care reported being able to have important conversations with their child. In the groups where no palliative care was received or uncertainty prevailed, the corresponding numbers were 40% and 33%, respectively. Around a third of the parents in the group whose child received palliative care (35%) claimed they talked with their child about death, while 19% of those whose child did not receive palliative care and 5% of those who were uncertain reported the same (**Tables 1**, **2**).

**Table 2:** Factors related to parental perception of the child’s care in the last month of life.

	Yes, my child received PC, n (%)	No, my child did not receive PC, n (%)	I am uncertain if my child received PC, n (%)
**Did your child receive palliative care (PC)?**	160 (70)	47 (21)	19 (9)
Do you think you could influence decisions about your child’s care?			
Completely agree	55 (34)	9 (19)	4 (21)
Mostly agree	60 (38)	16 (34)	6 (32)
Slightly agree	33 (21)	10 (21)	5 (26)
Completely disagree	11 (7)	9 (19)	3 (16)
*Missing*	1 (1)	3 (6)	1 (5)
Do you think that the healthcare professionals reacted quickly when your child needed help?			
Completely agree	93 (58)	18 (38)	11 (5)
Mostly agree	48 (30)	16 (34)	5 (26)
Slightly agree	12 (8)	6 (13)	1(5)
Completely disagree	5 (3)	5 (11)	2 (10)
*Missing*	2 (1)	2 (4)	–
Were you confident that your child would immediately receive help at home if needed?			
Completely agree	43 (27)	9 (19)	5 (26)
Mostly agree	53 (33)	6 (13)	4 (21)
Slightly agree	29 (18)	8 (17)	3 (16)
Completely disagree	14 (9)	7 (15)	2 (11)
*Missing*	19 (12)	15 (32)	3 (16)
Was your child admitted to the hospital if needed?			
Completely agree	107 (67)	34 (72)	14 (74)
Mostly agree	26 (16)	7 (15)	2 (11)
Slightly agree	6 (4)	2 (4)	1 (5)
Completely disagree	2 (1)	2 (4)	1 (5)
*Missing*	16 (10)	2 (4)	1 (5)
Did you feel that the healthcare professionals were competent?			
Completely agree	86 (54)	16 (34)	11 (58)
Mostly agree	52 (33)	21 (45)	5 (26)
Slightly agree	14 (9)	7 (15)	1 (5)
Completely disagree	4 (3)	3 (6)	2 (11)
*Missing*	4 (3)	–	–
Were you able to talk to your child about what was important?			
Yes, one time	10 (6)	1 (2)	2 (11)
Yes, several times	89 (56)	18 (38)	4 (21)
No, never	57 (36)	24 (51)	12 (63)
*Missing*	4 (3)	4 (9)	1 (5)
Did you talk to your child about death?			
Yes	56 (35)	9 (19)	1 (5)
No	100 (63)	32 (68)	16 (84)
*Missing*	4 (2)	6 (13)	2 (11)
Did you talk about the child’s impending death with anyone?			
Yes, one time	18 (11)	1 (2)	3 (16)
Yes, several times	107 (67)	16 (34)	5 (26)
No, never	35 (22)	28 (60)	9 (47)
*Missing*	–	2 (4)	2 (11)

### Factors related to the child and the parent’s perception of the child’s symptoms during the last month of life

According to the parents’ reports for children who received palliative care, the most common diagnosis was a brain tumor (45%), followed by children with leukemia (19%), sarcoma (10%), and other diagnoses (25%). In the two other groups of parental answers, leukemia was predominant. The malignant disease was identified as the leading cause of death in the group receiving palliative care (90%) and the group of uncertain parents (68%). Treatment-related complications represented the leading cause of death (49%) in the group who received no palliative care. Regardless of whether the child received palliative care or not, parents reported that during the last month of the child’s life, the child experienced multiple symptoms. In all three groups, the majority of parents reported that their child suffered to a large extent from physical weakness. In the group who received palliative care, the children suffered to a large extent from pain (64%) and poor appetite (61%) and were bothered by depression (32%), worry and anxiety (27%). When no palliative care was received the children were bothered to a large extent from poor appetite (54%), anxiety (43%), and respiratory distress (41%). Parents who were uncertain if their child received palliative care or not reported that the children to a large extent suffered from poor appetite (56%), pain (55%), weight loss (47%), and vomiting (41%) (**Tables 3**, **4**).

**Table 3:** Factors related to the child.

	Yes, my child received PC, n (%)	No, my child did not receive PC, n (%)	I am uncertain if my child received PC, n (%)
**Did your child receive palliative care (PC)?**	160 (70)	47 (21)	19 (9)
Child’s age			
Mean age (SD) at diagnosis	7.9 (5.20)	6.6 (5.55)	5.5 (4.93)
Mean age (SD) at death	10.6 (6.36)	9.1 (7.16)	8.5 (6.51)
Child’s sex			
Female	66 (42)	26 (55)	9 (47)
Male	94 (59)	21 (45)	10 (53)
Diagnosis			
Brain tumor	72 (45)	11 (21)	3 (16)
Leukemia	31 (19)	21 (45)	8 (42)
Skeletal sarcoma	16 (10)	1 (2)	3 (16)
Soft tissue sarcoma	12 (8)	-	-
Lymphoma	6 (4)	-	-
Neuroblastoma	7 (4)	4 (9)	2 (11)
Other malignant disease	14 (9)	9 (19)	2 (11)
*Missing*	2 (1)	1 (2)	1 (5)
Relapse			
One time	51 (32)	10 (21)	1 (5)
Several times	27 (17)	5 (11)	4 (21)
No remission of the primary diagnosis	80 (50)	29 (62)	9 (47)
*Missing*	2 (1)	3 (6)	5 (26)
Did the child express any wishes for their preferred location of death?			
No personal wishes	125 (78)	43 (91)	18 (95)
At home	22 (14)	-	–
At hospital	4 (3)	-	–
At another place	3 (2)	1 (2)	–
*Missing*	5 (3)	3 (6)	1 (5)
Cause of death			
The malignant disease	144 (90)	18 (38)	13 (68)
Treatment related complications	12 (8)	23 (49)	2 (11)
Other causes	4 (3)	3 (6)	3 (16)
*Missing*	-	3 (6)	1 (5)

**Table 4:** Factors related to the parent’s perception of the child’s symptoms the last month of life.

	Yes, my child received PC, n (%)	No, my child did not receive PC, n (%)	I am uncertain if my child received PC, n (%)
**Did your child receive palliative care (PC)?**	160 (70)	47 (21)	19 (9)
To what extent was your child bothered by physical weakness?			
	156	45	17
Large extent	127 (81)	27 (60)	10 (59)
Moderate extent	17 (11)	11 (24)	2 (12)
Little extent	7 (5)	2 (4)	2 (12)
Not at all	0 (0)	0 (0)	1 (6)
Not Applicable	5 (3)	5 (11)	2 (12)
To what extent was your child bothered by pain?			
	157	43	18
Large extent	100 (64)	16 (37)	10 (55)
Moderate extent	29 (18)	10 (21)	4 (22)
Little extent	17 (11)	6 (13)	3 (17)
Not at all	8 (5)	5 (11)	0 (0)
Not Applicable	3 (2)	7 (15)	1 (6)
To what extent was your child bothered by poor appetite?			
	155	44	16
Large extent	98 (63)	24 (54)	9 (56)
Moderate extent	27 (17)	5 (11)	2 (12)
Little extent	13 (8)	7 (16)	0 (0)
Not at all	9 (6)	4 (9)	1 (6)
Not Applicable	8 (5)	4 (9)	4 (25)
To what extent was your child bothered by nausea?			
	155	44	18
Large extent	78 (50)	14 (32)	6 (33)
Moderate extent	35 (23)	12 (27)	3 (17)
Little extent	19 (12)	10 (23)	4 (22)
Not at all	14 (9)	4 (9)	3 (17)
Not Applicable	10 (6)	4 (9)	2 (11)
To what extent was your child bothered by weight loss?			
	156	43	17
Large extent	59 (38)	13 (30)	8 (47)
Moderate extent	32 (20)	4 (9)	1 (6)
Little extent	31 (20)	9 (21)	2 (12)
Not at all	25 (16)	10 (23)	3 (18)
Not Applicable	9 (6)	7 (16)	3 (18)
To what extent was your child bothered by difficulty swallowing?			
	156	45	17
Large extent	54 (35)	12 (27)	2 (12)
Moderate extent	31 (20)	4 (9)	3 (18)
Little extent	23 (15)	6 (13)	5 (29)
Not at all	39 (15)	14 (31)	2 (12)
Not Applicable	8 (5)	8 (18)	5 (29)
To what extent was your child bothered by vomiting?			
	155	44	17
Large extent	53 (34)	11 (5)	7 (41)
Moderate extent	40 (26)	10 (23)	3 (18)
Little extent	27 (17)	12 (27)	0 (0)
Not at all	24 (15)	6 (14)	6 (35)
Not Applicable	11 (7)	5 (11)	2 (12)
To what extent was your child bothered by paralysis?			
	153	44	18
Large extent	54 (35)	4 (9)	5 (28)
Moderate extent	10 (6)	5 (11)	1 (6)
Little extent	20 (13)	5 (11)	1 (6)
Not at all	56(37)	21 (48)	8 (44)
Not Applicable	13 (8)	9 (20)	3 (17)
To what extent was your child bothered by depression?			
	155	45	18
Large extent	49 (32)	14 (31)	5 (28)
Moderate extent	40 (26)	7 (16)	6 (33)
Little extent	36 (23)	7 (16)	2 (6)
Not at all	21 (13)	8 (18)	3 (17)
Not Applicable	9 (6)	9 (20)	2 (6)
To what extent was your child bothered by worry and anxiety?			
	154	44	17
Large extent	42 (27)	19 (43)	3 (18)
Moderate extent	42 (27)	6 (14)	5 (29)
Little extent	38 (25)	5 (11)	2 (12)
Not at all	24 (16)	10 (23)	3 (18)
Not Applicable	8 (5)	5 (11)	4 (23)
To what extent was your child bothered by respiratory distress?			
	152	44	17
Large extent	41 (27)	18 (41)	6 (35)
Moderate extent	31 (20)	4 (9)	2 (12)
Little extent	34 (22)	4 (9)	5 (29)
Not at all	37 (24)	12 (27)	3 (18)
Not Applicable	6 (4)	6 (14)	1 (6)

## Discussion

In this nationwide study, the majority of parents of children deceased from cancer or treatment related complications reported that they were aware that their child received palliative care and felt informed about the incurable disease within weeks up to months before the child died. In this group, the most common diagnosis was a brain tumor with a disease-related death, and the care was often received at home. In the group of parents who reported that their child did not receive palliative care most parents claimed that they were informed about the incurable disease hours or days before the child died. The most common diagnosis in this group was leukemia, with a treatment-related death, and the care was often received at the intensive care unit.

Families living in urban or rural areas, compared to sparsely populated areas, reported to a greater extent that their child had received palliative care. A majority of parents whose child received palliative care stated that the healthcare professionals were competent in caring for their child. This confidence in healthcare professionals was also present among many parents in the other two groups. Not surprisingly, it was more likely to have talked about death with the child if palliative care was received, compared to those who did not receive palliative care, or if the parents expressed uncertainty about palliative care involvement. In all three groups, many children were affected by both physical and mental symptoms during the last month of their life.

In line with previous research in Sweden on children with cancer (21), a brain tumor was the most common diagnosis when parents reported that their child received palliative care. If parents did not perceive ongoing palliative care, leukemia was the predominant diagnosis. The results indicate differences in the timing of communication regarding prognosis between these two groups. Lack of timely communication is a well-known challenge in pediatrics (11). Parents who reported that their child received palliative care also reported that prognostic information was provided at an earlier stage of disease progression, compared to the group who did not receive palliative care. In the aforementioned group, one possible reason is that the child died from treatment-related complications, in line with the above-mentioned parental view, and therefore less likely to have had conversations about ‘disease incurability’. Another potential explanation is that the child’s disease progressed more rapidly, not leaving time for such conversations. Severe complications and rapid deterioration can lead to care in an intensive care unit, which may contribute to parents perceiving the treatment as curative. Previous research has shown that children with leukemia are more likely to die from complications related to treatment rather than to the disease itself, indicating high treatment intensity with an increased risk of complications (22).

Irrespective of the cancer diagnosis, prognosis is difficult to predict. A previous study has shown that pediatric oncologists are aware of the child’s potentially poor prognosis on average three months before the parents (23). Additionally, if both the parent and the physician recognize that there is no realistic chance of a cure, it is more likely that elements of palliative care will be included in the child’s care (23). According to the parents whose child received palliative care, the information about the incurable disease was given to most families with both parents present and sometimes also with the child present. This reflects an ‘open approach’ to disclosure of a prognosis and is based on the belief that children already know when they are seriously ill, and silence can create uncertainty and a lack of support for the dying child (24, 25). Research underscores the importance of balancing the complexity of these issues with considerations on a ‘case-by-case basis’, where aspects of the role of hope and culture are crucial (24). When the question was posed of whether the parent had talked to their child about death, the most common answer was ‘no’, regardless of whether the child had received palliative care. The literature confirms that it is an emotional and distressing situation and a major challenge for parents to convey difficult information and talk about death with their child (26). However, research has also shown that avoidance may increase the risk that parents regret their silence after the child’s death (6), and communication allows families to plan for their child’s final time and to help them cope with the severe circumstances (27). From the healthcare professionals’ perspective, studies highlight the fact that being a messenger of malignant disorders, communicating a poor or uncertain prognosis, especially to adolescents, is a challenging task (28, 29). When breaking bad news, physicians need to be open about the facts, be honest with their message, and at the same time convey hope (29, 30). Healthcare professionals state that providing care for the suffering and dying child adds complexity to all other challenges in end-of-life care (31, 32). Additionally, in the encounter between the family and the multidisciplinary healthcare providers, cultural issues, communication patterns and preferences need to be considered when disclosing bad news (24, 33). An early involvement of palliative care can help families and oncologists navigate the complexities and the ‘case-by case’ considerations (3).

In the present study, the child’s home was the most common place of death when receiving palliative care, and this has also been found to be the preferred location of care and death by parents and pediatric oncologists (34). Regardless of whether the child received palliative care, parents reported that they felt they had influence on decisions regarding their child’s care and they were confident that the child would get a place in the hospital and immediate help at home if needed. However, access to palliative care was not as good for families living in sparsely populated areas. A conceivable explanation is that the decentralized Swedish healthcare model plays a role and that regional differences become apparent when a child requires palliative care (13, 14). It should be noted that the study had a broad definition of palliative care and did not determine whether the care was provided by specialized or primary palliative care providers, nor did it identify the specific healthcare needs of each child. Despite differences in the level of care needed, most parents did not feel burdened by their child’s care, which aligns with a previous publication in the childhood cancer field (35). Practical support from healthcare professionals in managing daily tasks, along with parents’ perceptions that they are not obligated to take on more responsibilities than they can handle or wish to accept, is essential in this challenging situation, as noted by parents of children with serious illnesses (36). Irrespective of the type of care, the majority of parents reported that they considered the healthcare professionals competent. A contributing factor to their professional competence could be the specialized training in pediatric oncology that has been ongoing since the early 2000s in Sweden. These training programs for physicians and nurses include teaching and seminars on symptom control, communication, and palliative care, based on research and clinical experience. A scientific evaluation has been conducted regarding this training for nurses (37).

Parents reported that in the last month of the child’s life the child had physical weakness, pain and decreased appetite, and signs of depression and distress, irrespective of whether the child received palliative care. Regarding the symptoms affecting the children receiving palliative care, our findings do not differ from previous studies (38, 39). When children receive palliative care, it is part of the treatment to regularly assess various symptoms, particularly pain. This study did not explore symptom assessment; however, if the care is defined as palliative, it is likely that parents are particularly vigilant for signs that indicate a need for symptom relief. Regardless of how the form of care was defined, it was unsatisfactory to observe that not all children could have their symptoms alleviated. A recently published study shows that children’s symptoms need to be addressed more effectively (40). It is known that parents rate their child’s suffering higher than the child does themselves (41). Interestingly, when children with cancer report on their situation in their final days, their physical suffering is greater than their social and existential challenges (42).

The strength of this study is the national approach, including parental perceptions of their child’s incurable illness after treatment for several different cancer diagnoses. A limitation is the risk of recall bias regarding parental perceptions. Furthermore, we do not know if parents who reported that their child did not receive palliative care or those who were uncertain had wished to get this care for their child or received ‘non- articulated’ palliative care. The study has a response rate of less than 50%, and there is a potential risk for selection bias, as well as recruitment bias. However, given the subject area and the extensive study design with multiple research questions, the study findings are considered to reflect the target population.

In conclusion, understanding parental perceptions in pediatric palliative oncology care is vital for developing child-centered care. The study findings highlight the role of initiating palliative care early, the need for access to national equitable palliative care, and professional competence across the entire lifespan, regardless of diagnosis and place of residence.

## Data Availability

The raw data supporting the conclusions of this article will be made available by the authors upon reasonable request, without undue reservation.
